# The Sesquiterpene Lactone Cynaropicrin Manifests Strong Cytotoxicity in Glioblastoma Cells U-87 MG by Induction of Oxidative Stress

**DOI:** 10.3390/biomedicines10071583

**Published:** 2022-07-02

**Authors:** Rossella Rotondo, Maria Antonietta Oliva, Antonietta Arcella

**Affiliations:** I.R.C.C.S Istituto Neurologico Mediterraneo Neuromed, Via Atinense, 18, 86077 Pozzilli, Italy; rossellaross1988@gmail.com (R.R.); mariaantonietta.oliva@neuromed.it (M.A.O.)

**Keywords:** cynaropicrin, sesquiterpene lactone, oxidative stress, ROS, apoptosis, autophagy

## Abstract

Cynaropicrin has shown a wide range of pharmacological properties, such as antitumor action. Here, we showed the inhibitory effect of Cyn on human glioblastoma cell U-87 MG growth. According to the IC50 values, Cyn 4, 8 and 10 µM displayed a significant cytotoxicity, as confirmed by the cell count and MTT assay. Furthermore, Cyn completely abolished the ability of U-87 MG to form colonies and induced drastic morphological changes. Interestingly, pretreatment with ROS scavenger N-acetylcysteine 3 mM reversed the cytotoxicity induced by Cyn 25 µM and preserved the cells by morphological changes. Therefore, oxidative stress induction was evaluated at low 8- and high 25-µM concentrations in U-87 MG, as demonstrated by the quantitative and qualitative analysis of ROS. A prolonged increase in ROS generation under Cyn 25 µM exposure was followed by the loss of the mitochondrial membrane potential in treated U-87 MG cells. An acute treatment with Cyn 25 µM induced Cyt c release, as revealed by immunofluorescence staining and the activation of cell death pathways, apoptosis and autophagy. On the other hand, chronic treatment with Cyn 8 µM induced senescence, as revealed by the increase in SA-β-Gal activity. Moreover, at this concentration, Cyn led to ERK dephosphorylation accompanied by a relevant reduction of the NF-κB p65 subunit. Finally, the combined effect of TMZ and Cyn resulted in synergistic cytotoxicity, as evaluated by the Bliss additivity model. The strong cytotoxicity of Cyn was also confirmed on IDH1 mutant U-87 MG cells and patient-derived IDH wild-type glioblastoma cell lines NULU and ZAR. In conclusion, given the high toxicity at minimal concentrations, the high inhibition of tumor cell growth and synergy with the standard drug for glioblastoma TMZ, Cyn could be proposed as a potential adjuvant for the treatment of glioblastoma.

## 1. Introduction

Sesquiterpene lactones (SLs) are a wide group of structurally diverse secondary metabolites, commonly found in *Asteraceae* family plants [1,2]. SLs consist of 15 carbon compounds, derived from three isoprene units, rearranged in different cyclical conformations fused with a lactone group. Particular interest as antitumor agents has been attributed to the SLs of the guaianolide group. Among these, Cynaropicrin (Cyn), a major component of common artichoke Cynara *scolimus* L., has shown a wide range of pharmacological properties, such as antihyperlipidemic, anti-trypanosomal, antimalarial, antispasmodic and, antiphotoaging, as well as anti-inflammatory activity, associated with the suppression of the key proinflammatory NF-κB pathway [1,2,3,4,5,6,7]. Recently, Cyn showed a strong inhibitory effect on colorectal cancer in vitro and in vivo [8,9] and a potent cytotoxic effect on human melanoma cells A375 [10] and the HeLa cervical cancer cell line [11]. 

As reported for many SLs, the nucleophilic α, β-unsaturated carbonyl group of the lactone ring is able to form covalent bonds with sulfhydryl groups of several biomolecules via a Michael addition [12]. The direct consequence of these features is the alteration of intracellular redox homeostasis, leading to the excessive exposure of cancer cells to reactive oxygen species (ROS), though the blockage of antioxidant defense systems that are generally upregulated prevent oxidative damage [13]. It is known that radiation and chemotherapeutic drugs generate ROS during the treatment of cancer patients. Enhanced oxidative stress alters the cellular redox equilibrium, reactivating homeostatic mechanisms such as apoptosis and autophagy [14]. For this reason, recently, oxidative stress and the application of ROS-inducing compounds as adjuvant therapy have assumed a central role in glioblastoma (GBM) [15]. Despite numerous efforts to improve the prognosis, GBM, one of the most malignant neoplasms of the central nervous system (CNS) due to its rapid growth and infiltration, remains the most common brain tumor in adults, with a median survival of 15 months [16]. The standard protocol of glioblastoma patients provides surgery followed by chemoradiotherapy with alkylating agent Temozolomide (TMZ), the so-called Stupp protocol. These standard treatments, unfortunately, inefficiently control the growth of tumors that, after the period of time variable from patient to patient, recurs [15]. To complicate the findings of a resolutive therapy contributes an innate or acquired resistance to TMZ due to the methylation status of the O^6^-methylguanine methyl transferase (MGMT) gene promoter and the extreme heterogeneity of this tumor [16,17,18]. Indeed, the molecular characterization of brain tumors led to mainly classifying GBMs according to isocitrate dehydrogenase 1/2 (IDH 1/2) mutations and to establishing a correlation with MGMT gene promoter methylation [19,20]. However, despite the intriguing molecular classification, which contributes to the stratification of new entities within the same niche, novel therapies are urgently needed. In this context, natural compounds that can overcome the TMZ resistance and enhance its efficacy, without side effects at a higher dosage, have proven to be promising adjuvant therapies. In particular, a new strategy aims to re-sensitize the GBM response to TMZ by generating excessive ROS production, inducing cell death programs such as apoptosis and autophagy [21]. 

Finally, several SLs have been reported as promising molecules able to pass the blood–brain barrier, one of the main obstacles in the fight against glioblastoma [22,23,24]. 

This study will investigate the Cyn-induced effects in glioblastoma cells and the potential synergy with the conventional chemotherapy TMZ.

## 2. Materials and Methods

### 2.1. Materials

All reagents and chemicals (e.g., MTT assay or (3-(4,5-dimethylthiazol-2-yl)-2,5-diphenyltetrazolium), isopropanol, N-acetyl-cysteine, Tween-20, Triton X-100, 2′,7′-Dichlorofluorescin diacetate, ethylenediaminetetraacetic acid disodium salt dihydrate, EDTA and BSA) were of analytical grade (Sigma-Aldrich/Merck Life Science S.r.l., Milano, Italy). Nonfat dried milk powder was from PanReac (AppliChem ITW Reagents, Darmstadt, Germany). JC-1 dye (Mitochondrial Membrane Potential Probe) was from Invitrogen (Carlsbad, CA, United States). The 10% neutral-buffered formalin was purchased from Diapath (Diapath, Martinengo, Italy). Cynaropicrin ([(3*a*R,4S,6*a*R,8S,9*a*R,9*b*R)-8-hydroxy-3,6,9-trimethylidene-2-oxo-3*a*,4,5,6*a*,7,8,9*a*,9*b*-octahydroazuleno [4,5-*b*]furan-4-yl] 2-(hydroxymethyl)prop-2-enoate) with HPLC grade purity ≥ 95% was from Extrasynthese (Genay, Cedex, France). All reagents for the cell culture (e.g., DMEM, FBS, streptomycin/penicillin and PBS) were from EuroClone (Milan, Italy).

### 2.2. Cell Culture

Human continuous glioblastoma cell line U-87 MG was purchased from the Sigma Aldrich Collection (LGC Promochem, Teddington, UK). U-87 MG cells were cultured in Dulbecco’s Modified Eagle’s Medium supplemented with 10% fetal bovine serum (FBS), 2 mmol/L L-glutamine, 100 IU/mL penicillin, 100 µg/mL streptomycin at 37 °C, 5% CO_2_ and 95% humidity. The IDH1 mutant U-87 Isogenic Cell Line is a glioma IDH1R132H mutant isogenic line derived from the parental U-87 MG (ATCC HTB-14) cell line. The c.395G > A is a heterozygous mutation encoding IDH1R132H protein expression, generated at the ATCC (Manassas, VA, USA) by utilizing the CRISPR/Cas9 gene editing technology. This cell line was cultured in Eagle’s Minimum Essential Medium (EMEM) with 10% FBS and 100 IU/mL penicillin, 100 µg/mL streptomycin at 37 °C, 5% CO_2_ and 95% humidity. Primary human glioblastoma cell lines were established from the tumor biopsies of patients who gave their informed consent to participate in the study. The use of primary cell lines, as a model for GBM heterogeneity, was approved by the Ethics Committee on 27 February 2020 and registered on ClinicalTrials.gov with the identification number NCT04180046. Patient-derived wild-type IDH glioblastoma cell lines NULU and ZAR were characterized as previously reported [25]. In detail, primary glioblastoma cells NULU and ZAR were cultured in Dulbecco’s Modified Eagle’s Medium (DMEM) supplemented with 10% fetal bovine serum (FBS), 2 mmol/L-glutamine, 100 IU/mL penicillin, 100 μg streptomycin at 37 °C, 5% CO_2_ and 95% humidity.

### 2.3. IC50 Values Estimation

The half-maximal inhibitory concentration (IC50) values of Cyn at 24, 48 and 72 h on the human GBM continuous cell line U-87 MG and IDH1 mutant U-87 isogenic cell line were estimated by culturing 5 × 10^3^ cells/well in 96-well plates. The IC50 values were determined treating cells with 0.01, 0.1, 1, 10, 25, 50 and 100 µM and DMSO 0.1% as the vehicle control, respectively. The effects of Cyn on mitochondrial dehydrogenase activities to oxidize the tetrazolium into formazan salts were evaluated by the MTT assay. MTT 5 mg/mL were dissolved in phosphate-buffered saline (PBS), and 10 µL of the solution was added in each well. Formazan crystals were dissolved in an acidic isopropanol solution and the absorbance measured at 595 nm. The IC50 values were estimated with GraphPad Prism 7 software (GraphPad Software Inc., San Diego, CA, USA).

### 2.4. Cell Proliferation and Cytotoxicity Assays

The long-term effects of Cyn treatment on U-87 MG cells were evaluated by cell proliferation and cytotoxicity assays. The proliferation of cells under Cyn exposure was assessed by plating 1 × 10^4^ U-87 MG cells in 48-well plates. On the basis of the IC50 values, the cells were treated daily with Cyn 4, 8 and 10 µM and DMSO 0.1% for 24, 48 and 72 h, with the cell counts performed at the same time points. Changes in the mitochondrial dehydrogenase activities in glioblastoma cells were evaluated by the MTT assay by plating 5 × 10^3^ cells/well in 96-well plates. In detail, U-87 MG cells and patient-derived glioblastoma cell lines NULU and ZAR were treated daily with Cyn 4, 8 and 10 µM and DMSO 0.1% for 24, 48 and 72 h. At each time point, 10 µL of MTT (5 mg/mL in PBS) were added per well and formazan crystals dissolved, as reported in Section 2.3.

### 2.5. Colony Forming Assay

U-87 MG cells (10^3^ cells/well) were seeded in a 6-mutiwell plate in DMEM with 10% FBS for 48 h. Cells were treated with Cyn 4 μM and the control vehicle (0.1% DMSO) for 24 h, and the medium was replaced every 3 days for 14 days. The colonies were fixed with 4% paraformaldehyde solution for 5 min, washed with PBS and stained with crystal violet 0.05% for 30 min.

### 2.6. Pretreatment with N-Acetylcysteine

Cyn-induced oxidative stress in U-87 MG cells was assessed by plating 5 × 10^3^ cells in 96-multiwell plates in DMEM with FBS 0.5% for 48 h. Cells were then preincubated with N-acetylcysteine (NAC) 3 mM for 4 h at 37 °C. After 4 h, the medium was replaced and U-87 MG cells treated with different concentrations of Cyn (0.01, 0.1, 1, 10, 25, 50 and 100 µM) for 24 h. DMSO 0.1% was used as the vehicle control. After treatment, morphological changes of U-87 MG were observed and microscopically imaged (Evos, Life technologies, Carlsbad, CA, USA). The inhibition of the oxidative stress-induced cytotoxicity of Cyn was evaluated by the MTT assay performed as reported in Section 2.3.

### 2.7. Quantitative and Qualitative Assessment of Reactive Oxygen Species (ROS) Generation by H2DCFDA

ROS generation was detected by using the cell-permeable fluorogenic probe 2′,7′-dichlorodihydrofluorescein diacetate (H2DCFDA). U-87 MG cells were plated in 60-mm cell culture plates at density of 5.0 × 10^5^ cells in DMEM with FBS 10%. The medium was replaced with DMEM with FBS 0.5% for 48 h. U-87 MG cells were treated with Cyn 8 and 25 μM for 2 and 6 h. Cells treated with DMSO 0.1% were used as the control. At the end of the treatments, cells were incubated with H2DCFDA 50 µM for 30 min at 37 °C in the dark. Cells were then washed with PBS, scraped in 100 μL PBS with 2% Triton X-100 and lysates were collected in a 1.5-mL tube on ice in the dark. Cells without H2DCFDA incubation were collected to measure the cellular autofluorescence. Samples were centrifuged at 14,000 rpm for 15 min at 4 °C. The conversion of nonfluorescent H2DCFDA to fluorescent 2′,7′-dichlorofluorescein (DCF) by Cyn-induced ROS was estimated by a microplate reader (Ex/Em = 488/525 nm) and data reported as the mean ± SEM of three determinations. For the qualitative assessment, the cells plated in 12-multiwell plates at a density of 1.0 × 10^4^ and were observed under the EVOS^®^ FL Cell Imaging System (Life Technology, Carlsbad, CA, USA).

### 2.8. Qualitative and Quantitative Analysis of Mitochondrial Transmembrane Potential Changes (ψm) with JC-1

The qualitative mitochondrial membrane potential (ΔΨm) in U-87 MG cells was assessed by the EVOS^®^ FL Cell Imaging System (Life Technology, Carlsbad, CA, USA) through 5,5′,6,6′-tetrachloro-1,1′,3,3′-tetraethyl-imidacarbocyanine iodide (JC-1; Molecular Probes, Invitrogen) following the manufacturer’s instructions. In detail, 1.0 × 10^4^ U-87 MG cells were seeded in a 48-multiwell plate in DMEM with 10% FBS. The medium was replaced with DMEM with FBS 0.5% for 48 h to synchronize the cells. Treatments were performed using Cyn 25 µM and DMSO 0.1% as the control for 24 h. For the positive control, U-87 MG cells were incubated with 10 μM H_2_O_2_ for 30 min. At the end of each treatment, the cells were washed with PBS and incubated with 10 µg/mL JC-1 in DMEM without FBS for 20 min at 37 °C in the dark. Before being used, the JC-1 solution was sonicated in order to disaggregate the crystals. A qualitative analysis of the progressive loss of red J-aggregate fluorescence and the cytoplasmic diffusion of green monomer fluorescence, following the exposure of cells to Cyn, H_2_O_2_ and DMSO, were assessed, imaging the cells in the red/green channels. For a quantitative analysis, change in the red/green fluorescence ratio was evaluated for single cell by ImageJ software and data reported as the mean ± SEM of 3 individual determinations.

### 2.9. Immunofluorescence for NRF2 Traslocation and Cytocrome c Release

The nuclear translocation of Nuclear factor erythroid 2-related factor 2 (Nrf2) and release of Cytochrome c (Cyt c) from mitochondria under Cyn treatment were evaluated by immunofluorescence staining. In detail, U-87 MG cells were seeded at density of 1.0 × 10^4^ in an 8-multiwell chamber in DMEM with 10% FBS. The medium was replaced with DMEM with 0.5% FBS for 48 h. Cells were than treated with Cyn 25 μM for 24 h and DMSO 0.1% as the control in DMEM with 10% FBS. At the end of the treatment, cells were washed twice with phosphate-buffered saline (PBS) and fixed in 4% formalin for 20 min and permeabilized with 0.1% Triton for 30 min. After blocking with 10% specific serum, the cells were incubated with antibody against Nrf2 (Ab89443, Abcam, Burkingane, CA, USA, 1:100) and Cyt c (BD Pharmingen™, San Diego, CA, USA, 1:100) overnight at 4 °C. After washing with 0.025% PBS–Tween-20, the cells were incubated with secondary antibody anti-mouse fluorescein (1:100; Vector, Stuttgart, Germany) in 2% serum for 1 h at room temperature. The slides were counterstained with DAPI mounting medium (Vectashield, Vector Laboratories, Burlingame, CA, USA) for nuclei detection with an EVOS FL microscope at 60× magnitude.

### 2.10. DNA Ladder

U-87 MG cells were plated (4.5 × 10^5^) in DMEM with 0.5% FBS for 48 h and incubated with Cyn 25 µM and DMSO 0.1% as the vehicle control for 24 and 72 h. At the end of the treatment, the cells were collected, and the pellets were washed with PBS. DNA was extracted with the QIAamp DNA Blood Mini kit (Qiagen, Hilden, Germany), and the DNA fragments were separated by 2.5% agarose gel electrophoresis and visualized by SYBR^TM^ Safe DNA Gel Stain (Invitrogen, Life Technologies, CA, USA).

### 2.11. SA-β-Gal Activity Assay

Cyn-induced senescence was evaluated by seeding cells at a density of 1 × 10^4^ U-87 MG cells in an 8-multiwell chamber slide in DMEM with 0.5% FBS for 48 h. Cells at sub-confluency were treated daily with Cyn 8 µM and DMSO 0.1% in DMEM with 10% FBS for 24 and 72 h. At the end of each treatment, senescence induction was evaluated with the SA-β-Gal activity assay by using the Senescence Detection Kit (BioVision, Mountain View, CA, USA) following the manufacturer’s instructions. Briefly, U-87 MG cells were gently washed with PBS and subsequently fixed with fixative solution for 15 min at room temperature. After incubation with a staining solution containing 5-Bromo-4-Chloro-3-Indolyl β-D-Galactopyranoside (X-Gal), the percentual increase in senescent cells was microscopically evaluated, counting the number of SA-β-Gal-positive cells versus the number of total cells.

### 2.12. Cell Signaling Studies

To investigate Cyn-mediated cell signaling, 5 × 10^5^ U-87 MG cells were seeded in 60-mm plates in DMEM with 0.5% FBS for 48 h. The medium was then replaced and cells treated with Cyn at their corresponding apoptotic-inducing and senescence-inducing concentrations of Cyn for short- and long-term treatments. Protein extraction was performed in Tris-HCl 50 mM pH 8, EDTA 5 mM, NaCl 150 mM, 1% Triton X-100, protease (Santa Cruz Biotechnology, Dallas, TX, USA) and the phosphatase inhibitors (Roche Diagnostics GmbH, Mannheim, Germany). The protein concentrations were determined using the Bradford assay with Bio-Rad Protein Assay Dye Reagent Concentrate (Bio-Rad Laboratories, Inc., Hercules, CA, USA). Fifteen milligrams of proteins were loaded of each sample and sodium dodecyl sulfate polyacrylamide gel electrophoresis (SDS-PAGE) performed. The proteins were transferred onto a PVDF membrane (GE Healthcare Life Sciences, Little Chalfont, UK) for the appropriate time. Nonspecific binding sites were blocked with 5% (*w/v*) nonfat dry milk or BSA in Tris-buffered saline with Tween-20 0.1% (TBS-T) and membranes probed with primary specific antibodies overnight at 4 °C: Caspase 3 (Cell Signaling Technology, 1:1000), Caspase 9 (Cell Signaling Technology, 1:1000), LC3B (Cell Signaling Technology, 1:1000), p62 (Cell Signaling Technology, 1:1000) and NF-κB p65 subunit (Santa Cruz, 1:500). A specific secondary antibody (EMD Millipore Corp., 1:7000) was applied for 1 h at room temperature. β-actin (Sigma, 1:100,000) and GAPDH (Cell Signaling Technology, 1:1000) were used to normalize the levels of the proteins. For phospho-Thr202/Tyr204p44/42 MAPK (pErk1/2) (Cell Signaling Technology, 1:1000) after overnight incubation at 4 °C and signaling acquisition, the membrane was stripped and re-probed with p44/42 MAPK (Erk1/2) (Cell Signaling Technology, 1:1000) under the same conditions. All immunoblots were developed using the chemiluminescence (ECL) detection system (GE healthcare Life Sciences, Milan, Italy), while the digital signals were quantified by densitometric analysis using Image Lab software 6.1 for Windows (Bio-Rad Laboratories, Rome, Italy).

### 2.13. Synergistic Activity of Cynaropicrin and Temozolomide

The synergy between Cyn and TMZ was assessed by the Bliss additivity model [26]. Cell death was expressed as a fraction of the 0.1% DMSO-treated controls. For combination cytotoxicity experiments, the cells were exposed to a fixed concentration ratio of IC50 values for Cyn and TMZ (0.25, 0.5, 1, 2 times the IC50). After 48 h of drug exposure, the fraction of cells affected by each drug and the combination were calculated. The nature of the interactions between drugs (synergy/antagonism) was assessed at each drug dose using the Bliss additivity model by which the observed cytotoxic effects are compared to that theoretically predicted for the additive effects alone. Under this model, the predicted additive effect for two single compounds with effects A and B is ((A + B) − (A × B)), where each effect is expressed as a fractional inhibition between 0 and 1. Statistical significance of the interaction effect was formally tested using a Student’s *t*-test to assess the positive or negative deviation of the mean from zero.

### 2.14. Statistical Analysis

GraphPad Prism 7 software (GraphPad Software, Inc., La Jolla, CA, USA) was used for the statistical analyses. Data were reported as the mean ± SEM (*n* = 3). The statistical significance or variance was determined by using an unpaired Student’s *t*-test or one-way ANOVA, considering a *p*-value less than 0.05 statistically significant. According to GraphPad Prism 7 software, * a *p*-value 0.01–0.05 was considered statistically significant, ** a *p*-value 0.001–0.01 very significant and *** a *p*-value 0.0001–0.001 extremely significant, while **** a *p*-value < 0.0001 was very extremely significant.

## 3. Results

### 3.1. Cynaropicrin Induces Cytotoxicity in U-87 MG Glioblastoma Cell Line

**Figure 1:**
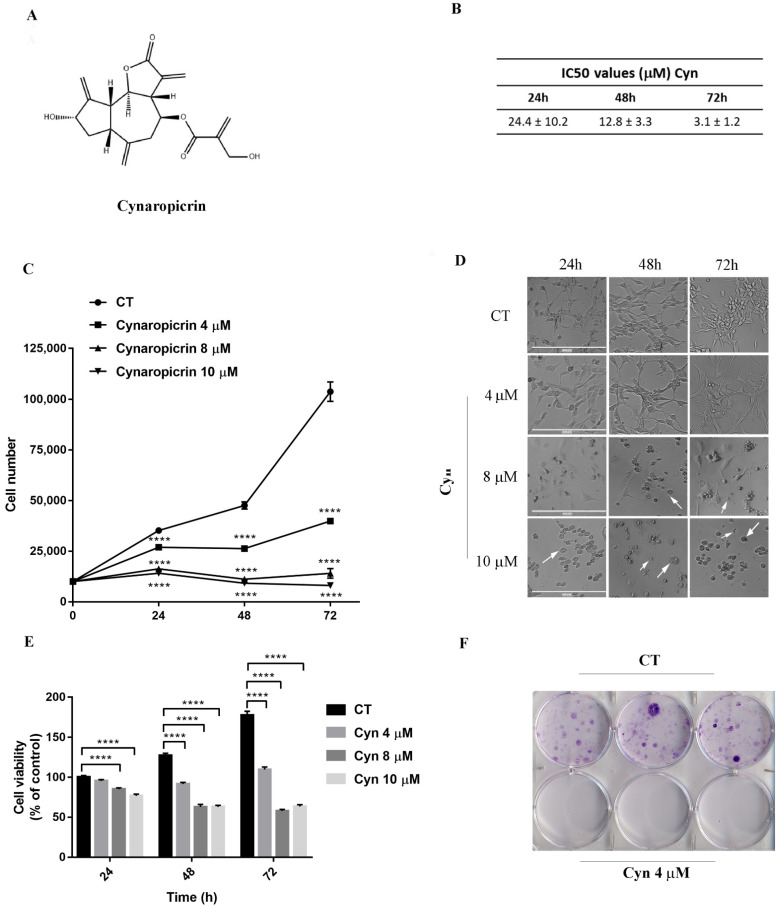
**Cytotoxic effects of Cyn on human glioblastoma U-87 MG cells.** (**A**) Molecular structure of SL Cynaropicrin (IUPAC name: ([(3*a*R,4S,6*a*R,8S,9*a*R,9*b*R)-8-hydroxy-3,6,9-trimethylidene-2-oxo-3*a*,4,5,6*a*,7,8,9*a*,9*b*-octahydroazuleno[4,5-*b*]furan-4-yl] 2-(hydroxymethyl)prop-2-enoate). (**B**) Determination of IC50 values on U-87 MG cells using GraphPad Prism 7 software after 24, 48 and 72 h of incubation with Cyn 0.01, 0.1, 1, 10, 25, 50 and 100 µM and DMSO 0.1% as the vehicle control. (**C**) Dose and time-dependent reduction of the U-87 MG cell number under daily exposure to Cyn 4, 8 and 10 µM for 24, 48 and 72 h. (**D**) Morphological change of 4, 8 and 10 µM Cyn-treated U-87 MG cells for 24, 48 and 72 h. White arrows indicate round-shaped cells with a loss of filaments and the presence of cell shrinkage. (**E**) Cytotoxic effects of daily exposure to Cyn 4, 8 and 10 µM for 24, 48 and 72 h on the U-87 MG cell metabolism. (**F**) Effect of Cyn 4 µM on the U-87 MG clonogenic potential. For all the experiments, values are the mean ± SEM of 3 individual determinations. One-way ANOVA test, *p*-value < 0.05. According to GraphPad Prism 7 software, **** *p*-value < 0.0001 (extremely significant).

The cytotoxicity of Cyn on U-87 MG glioblastoma cells was first evaluated by IC50 value estimations at different time points. This molecule exerted a strong time-dependent cytotoxic effect. Indeed, approximately 2- and 7.8-fold lower IC50 values were estimated, respectively, at 48 h and 72 h than the IC50 values at 24 h (24.4 ± 10.2 µM), as shown in Figure 1B. The effect on U-87 MG cell growth, which was treated daily with different concentrations of Cyn 4, 8 and 10 μM for 24, 48 and 72 h, was evaluated by cell counting. Cyn induced a time and dose-dependent reduction of the cell numbers: in particular, Cyn 8 and 10 μM reduced more than 50% of the cell number at 24 h. At same concentrations, a reduction of more than 70% and 90% was observed, respectively, at 48 and 72 h (Figure 1C). Cyn-induced cytotoxicity was supported by the change of the cellular conformation, already appreciable after 48 h of treatment with Cyn 8 μM (Figure 1D). In fact, U-87 MG cells appeared round-shaped, with a loss of filaments and cell shrinkage. Furthermore, the MTT assay confirmed the cytotoxic effects of Cyn, which interfered with the U-87 MG cell metabolism. Mitochondrial dehydrogenase activities were inhibited 50% when the cells were treated with Cyn 8 and 10 μM for 48 h. At the same concentrations, a residual metabolic activity of 30% was estimated in cells under Cyn exposure for 72 h (Figure 1E).

### 3.2. Cynaropicrin Abolished Clonogenic Capacity of U-87 MG Glioblastoma Cell Line

In order to assess the effect of Cyn on the clonogenic potential of U-87 MG cells, a colony-forming assay was performed. On the basis of cytotoxicity experiments (Figure 1E), GBM cells were treated with Cyn 4 μM for 24 h. As reported in Figure 1F, Cyn completely abolished the ability of U-87 MG cells to form colonies.

### 3.3. N-Acetylcysteine Reverses Cynaropicrin-Induced Cytotoxicity

**Figure 2:**
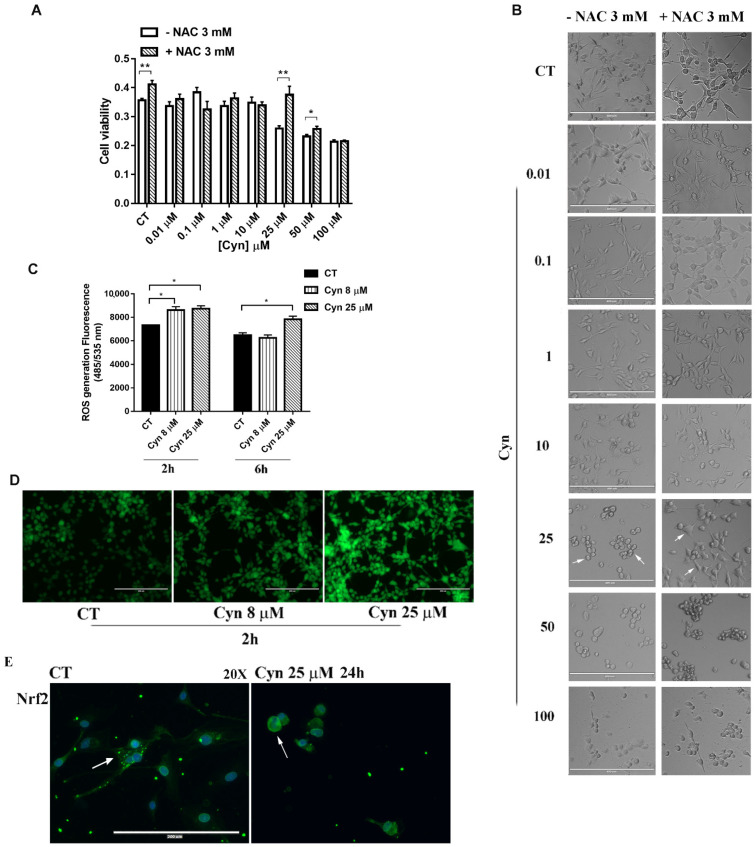
**Cyn-induced oxidative stress in human glioblastoma U-87 MG cells.** (**A**) Pretreatment of U-87 MG cells with NAC 3 mM for 4 h, followed by incubation for 24 h with Cyn 0.01, 0.1, 1, 10, 25, 50 and 100 μM. DMSO 0.1% was used as the vehicle control. (**B**) Preincubation with NAC 3 mM for 4 h preserved U-87 MG from Cyn-induced morphological change, since the cells maintained their protrusions rather than appeared round-shaped (white arrows). (**C**) Quantitative analysis of ROS generation in U-87 MG cells under 2 and 6 h of treatment with Cyn 8 and 25 μM. DMSO 0.1% was used as the vehicle control. (**D**) Qualitative analysis of ROS production after 2 h of exposure to Cyn 8 and 25 μM. (**E**) Immunofluorescence staining of NRF2 in U-87 MG treated for 24 h with Cyn 25 µM, which showed a nuclear localization with respect to the control cells treated with 0.1% DMSO (white arrows). Magnification 20×. Data were analyzed by a Student’s *t*-test, *p*-value < 0.05. According to GraphPad Prism 7 software, * *p*-values from 0.01 to 0.05 (significant) and ** *p*-values from 0.001 to 0.01 (very significant).

First evidence on the molecular mechanisms that underlined the Cyn-induced cytotoxicity was obtained by preincubating U-87 MG cells with antioxidant N-acetylcysteine (NAC) 3 mM for 4 h. NAC reversed the Cyn-mediated metabolic reduction, as revealed by the MTT assay. After 24 h of treatment with Cyn 25 μM and 50 μM, the cells preserved, respectively, 32% and 10% of the metabolic activities with respect to the NAC-untreated cells. However, in the same conditions, 100 μM Cyn-induced cytotoxicity exceeded the protective effect of NAC (Figure 2A). Additionally, NAC prevented cellular morphological change, since the cells treated with this ROS scavenger appeared elongated with a fusiform shape rather than round-shaped, as visible under the Cyn treatment (Figure 2B). Taking together, these results led us to suppose Cyn as a ROS-inducing compound in glioblastoma cells.

### 3.4. Cynaropicrin Increases ROS Generation, Alterates Mitocondrial Membrane Potential (Ψm) and Induces the Release of Cytocrome c

**Figure 3:**
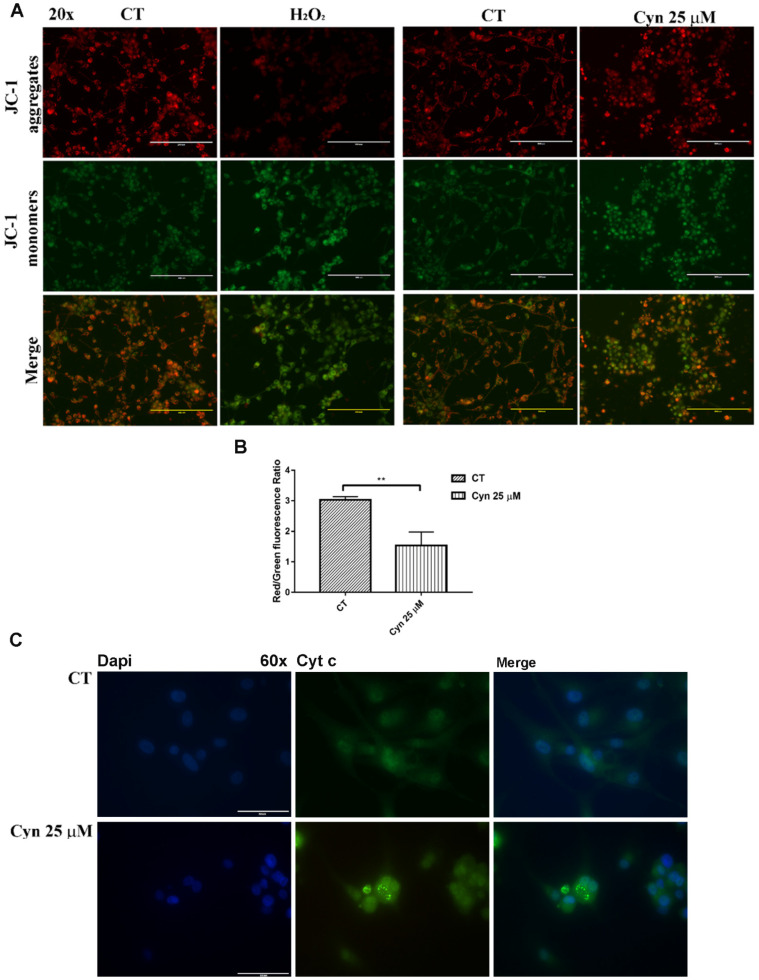
**Effects of the ROS-inducing compound Cyn on the mitochondria.** (**A**) Loss of the mitochondrial membrane potential (Ψm) in U87-MG cells treated with Cyn 25 µM for 24 h, evaluated by the JC-1 assay. DMSO 0.1% was used as the vehicle control, while exposure to H_2_O_2_ 10 µM for 30 min was the positive control. (**B**) Quantitative analysis of Ψm was estimated as a red/green fluorescence ratio in the same conditions using ImageJ software v. 1.53. Data were analyzed by the Student’s *t*-test, *p*-value < 0.05. According to GraphPad Prim 7 software, a ** *p*-value from 0.001 to 0.01 was considered very significant. (**C**) Immunofluorescence staining of Cyt c in 25 µM Cyn-treated U-87 MG cells for 24 h. DMSO 0.1% was used as the vehicle control. In U-87 MG cells treated with 25 µM Cyn for 24 h, Cyt c staining appeared much more punctate, a common feature indicative of mitochondrial fragmentation, rather than distributed throughout the cell body. Magnification 60×.

The assessment of ROS production in U-87 MG cells exposed to Cyn was confirmed by the quantitative analysis of fluorogenic dye H2DCFDA that measures hydroxyl, peroxyl and other ROS activity within the cell. According to a previous work in which Cyn was tested on HeLa cells [11], U-87 MG cells were treated for 2 and 6 h with different concentrations of Cyn. In particular, the level of ROS was evaluated treating cells with a Cyn 8 μM concentration that led to a statistically significant reduction of U-87 MG viability after 24 h of treatment and with a Cyn 25 μM concentration at which NAC protected cells from Cyn-induced cytotoxicity. As reported in Figure 2C, the quantitative analysis revealed that the treatment with increasing concentrations of Cyn significantly increased the ROS levels after 2 h of exposure. In accordance with the short half-life of ROS [27], after 6 h of Cyn exposure, the generation of ROS was still detectable at a higher concentration of Cyn (25 μM), but the initial burst of ROS was not maintained at the lowest concentration (8 μM) with respect to the control. The qualitative analysis confirmed ROS generation after 2 h of exposure to Cyn 8 and 25 μM by the increase in green fluorescent intensity compared with untreated cells, as shown in Figure 2D. Moreover, as a master regulator of oxidative stress, the nuclear translocation of Nrf2 was evaluated by immunofluorescence staining. In U-87 MG treated with Cyn 25 µM for 24 h, Nrf2 translocated from the cytoplasm to the nucleus (Figure 3E).

Rapid depolarization of the inner mitochondrial membrane potential (ΔΨm) induced by an increased level of ROS can be evaluated by JC-1 staining. Cyn 25 μM caused the loss of ΔΨm, determined by the inability of red J-aggregates to accumulate in the mitochondria, while diffusing as green fluorescent J-monomers when compared with the control (Figure 3A). The quantitative analysis confirmed that the ratio between the red and green fluorescence of JC-1 in U-87 MG cells treated with Cyn 25 μM led to the loss of the mitochondrial membrane potential (Figure 3B). Mitochondrial depolarization in Cyn-treated U-87 MG cells can alter the permeability of the transition pores of the mitochondrial membrane with the subsequent release of Cyt c in the cytosol, as it was visible in immunofluorescence staining in Figure 3C. This event, in turn, can trigger the activation of the deadly apoptotic pathway.

### 3.5. Cyn-Induced Acute Toxicity Mediates Apoptosis and Autophagy

**Figure 4:**
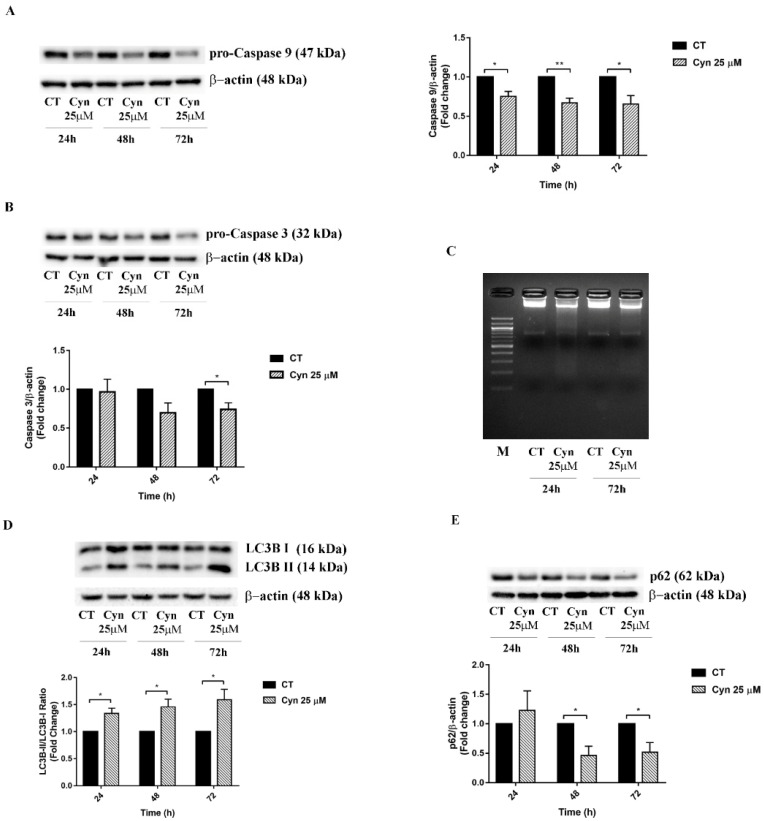
**Activation of U-87 MG cell death pathways under acute treatment with Cyn.** (**A,B**) Western blot analysis of pro-Caspase 9 and pro-Caspase 3 reduction in U-87 MG cells treated with Cyn 25 µM for 24, 48 and 72 h of exposure. Densitometric analysis of the protein levels represent the means ± SEM of 3 individual determinations. Data were normalized to housekeeping gene Actin and are expressed as a fold change over control treated cells. Statistical analysis was performed with the Student’s *t*-test, *p*-value < 0.05. According to GraphPad Prim 7 software, a ** *p*-value from 0.05 to 0.01 was considered significant and ** *p*-value from 0.001 to 0.01 very significant. (**C**) Agarose gel electrophoresis of DNA extracted from U-87 MG cells treated with Cyn 25 µM for 24 and 72 h. DMSO 0.1% was used as the vehicle control. (**D,E**) Western blot analysis of autophagic markers LC3B and p62. Densitometric analysis of protein levels represents the means ± SEM of 3 individual determinations. For LC3B, data were reported as a LC3B II/I ratio, while, for p62, the values were normalized to housekeeping gene Actin. The results are expressed as a fold change over control-treated cells. Statistical analysis was performed with the Student’s *t*-test, *p*-value < 0.05. According to GraphPad Prim 7 software, a * *p*-value < 0.05 was considered significant.

Since ROS has been previously reported as a possible mediator of apoptosis and autophagy [28], the observed results indicated the potential of Cyn in the activation of cell death pathways in U-87 MG-treated cells. In this context, the proapoptotic and pro-autophagic pathways were activated when U-87 MG cells were exposed to the acute toxicity of Cyn, as shown in Figure 4. According to the apoptotic cascade, the statistically significant reduction of pro-caspase 9 at 24, 48 and 72 h was followed by the activation of pro-caspase 3, which resulted in a significant decrease after 72 h of treatment with Cyn 25 μM (Figure 4A,B). Furthermore, the DNA fragmentation assay confirmed the apoptosis induction under Cyn 25 μM, as shown in Figure 4C, from the formation of a DNA ladder.

To determine whether a high dose of Cyn induced the autophagy, we assessed the expression of LC3B and p62 by Western blotting. Cyn treatment significantly increased the LC3B II/I ratio at 24, 48 and 72 h of exposure and dramatically reduced the expression level of p62 at 48 and 72 h.

### 3.6. Cyn-Induced Chronic Toxicity Induces Aging

**Figure 5:**
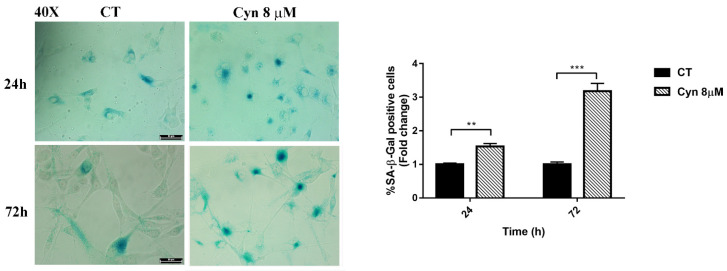
**Activation of senescence in U87MG under chronic treatment with Cyn.** Change in the SA-β-gal activity in U87MG cells treated daily with Cyn 8 µM for 24 and 72 h was reported as a percentage increase. Cells were microscopically evaluated, and the number of SA-β-Gal-positive cells versus the number of total cells was counted at 40× magnification. The results are expressed as a fold change over control-treated cells. Statistical analysis was performed with the Student’s *t*-test, *p*-value < 0.05. According to GraphPad Prim 7 software, a ** *p*-value from 0.001 to 0.01 was considered very significant and *** a *p*-value from 0.0001 to 0.001 was extremely significant.

Since marginal but continuous induction with low concentrations of ROS-inducing compounds has been reported to drive cellular senescence [29], the long-term effects of Cyn 8 μM on U-87 MG cells was evaluated. Considering that Cyn 8 μM induced a transient increase in the ROS levels compared with the higher concentration of Cyn 25 μM, as shown in Figure 2C, β-galactosidase (SA-β-gal) activity that correlates with the occurrence of senescence was evaluated [30]. Under this experimental condition, long-term treatment with a low dose of Cyn activated premature senescence. As shown in Figure 5 the percentage in SA-β-gal U-87 MG-positive cells increased from 1.5-fold at 24 h to 3-fold at 72 h. These results indicate that Cyn-induced ROS may represent a lead mechanism in the control of cell growth.

### 3.7. Short-Term Treatment of U-87 MG with Cynaropicrin Reduces ERK Phosphorilation and NF-κB Protein Expression

NF-κB and the MAPK signaling pathways have been ranked among the most common dysregulated pathways in glioblastoma cells, commonly implicated in the proliferation and survival of cancer cells [31,32].

**Figure 6:**
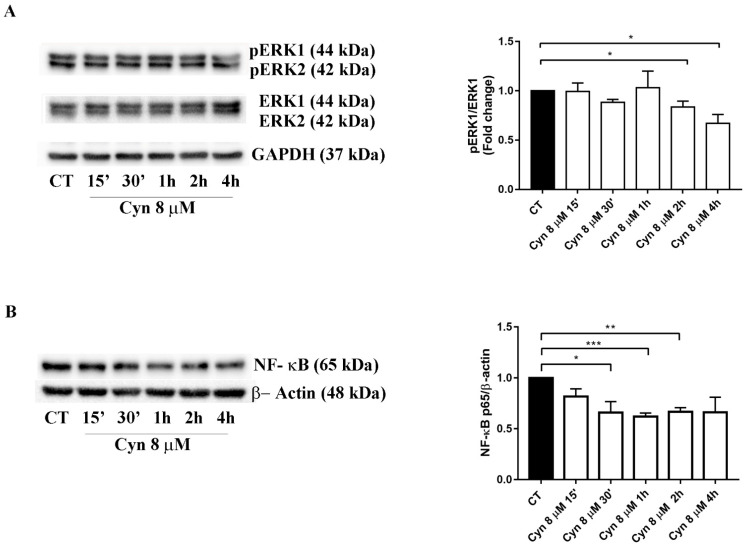
**Effects of Cyn on ERK1/2 dephosphorylation and NF-κB protein expression in U-87 MG cells treated with Cyn 8 µM.** (**A**) Western blot analysis of proliferative signals pERK/ERK in short-term U87Mg cells treated with Cyn 8 µM. A densitometric analysis of the protein levels represent the means ± SEM of 3 individual determinations and are expressed as the fold change over control-treated cells. * Unpaired *t*-test, *p*-value < 0.05. According to GraphPad Prism, * *p*-values 0.01–0.05 (significant). (**B**) Western blot analysis of the NF-κB p65 subunit in short-term U87Mg cells treated with Cyn 8 µM. A densitometric analysis of the protein levels represents the means ± SEM of 3 individual determinations. Data are expressed as the fold change over control-treated cells normalized to the housekeeping gene β-actin. * Unpaired *t*-test, *p*-value < 0.05. According to GraphPad Prism, * *p*-values 0.01–0.05 (significant), ** *p*-values 0.001–0.01 (very significant) and *** *p*-values 0.0001–0.001 (extremely significant).

Therefore, we evaluated if the ability of Cyn to decrease the proliferation of glioblastoma cells was associated with MAPK pathway regulation and, in particular, with ERK, the downstream effector of MAPK signaling. Our results confirmed that short-term treatment with Cyn 8 μM caused ERK dephosphorylation after 2 h and 4 h of exposure (Figure 6A).

NF-κB is a prominent antiapoptotic factor that induces the transcription of different antiapoptotic proteins. Protein expression of the NF-κB p65 subunit exhibited a strong significant reduction after 30 min until 2 h of exposure to Cyn 8 μM. This data correlated with the previous literature data that indicated NF-κB as the inhibitory target of Cyn [10,33].

### 3.8. Synergistic Effects of Cynaropicrin and Conventional Chemotherapy Temozolomide on U-87 MG Cells and Potential Use on IDH1-Mutant U-87 MG Cell Line and Patient-Derived Glioblastoma Cell Lines NULU and ZAR

**Figure 7:**
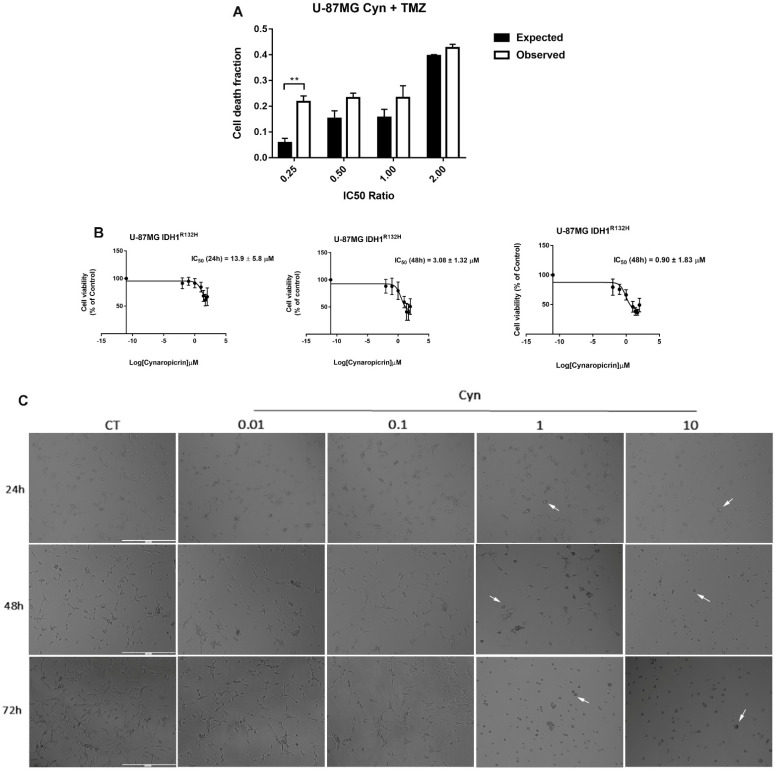
**Combined effects of Cyn and TMZ.** (**A**) Bliss additivity model to test the combined effects between Cyn and TMZ, using concentration ratios of 0.25×, 0.5×, 1× and 2× their IC50. Data are expressed as expected versus observed cell death fractions. Unpaired *t*-test, *p*-value < 0.05. According to GraphPad Prism 7 software,** *p*-value 0.001–0.01 (very significant). (**B**) Determination of IC50 values on IDH1 R132H U-87 MG cells using GraphPad Prism 7 software after 24, 48 and 72 h of incubation with Cyn 0.01, 0.1, 1, 10, 25, 50 and 100 µM and DMSO 0.1% as the vehicle control. (**C**) Morphological change of Cyn-treated IDH1 R132H U-87 MG cells for 24, 48 and 72 h. Cyn 1 µM dramatically induced a cell morphological change already visible after 24 h of exposure, since cells retracting their cell protrusions become round-shaped (white arrows). Magnification 10×.

Despite the intense research effort, radio-chemotherapy with Temozolomide remains the standard of care for glioblastoma treatment (Stupp protocol). In order to improve the efficacy of TMZ, we verified whether the combined effects of two agents, TMZ and Cyn, could result in synergistic cytotoxicity through Bliss additivity model. Therefore, combining the IC50 ratios of TMZ and Cyn, it has been assessed that a fractional inhibition of the observed cytotoxic effects exceeds the theoretically predicted cytotoxicity derived from the additive effects of TMZ and CYN as single agents (Figure 7A).

The newly updated 2021 WHO Classification of Tumors of the Central Nervous System distinguished glioblastoma in wild-type and mutant IDH1/2 [20].

Although it has been reported that patients with an IDH mutant glioblastoma and methylated MGMT gene promoter had longer survival than patients with IDH wild-type glioblastoma and an unmethylated MGMT gene promoter [19]; their prognosis remains poor. The IDH1 R132H is among the most common mutations in glioma that serves as an important diagnostic and prognostic marker in patients. This cell line has been tested at the ATCC for neomorphic activity of the mutant IDH1 R132H enzyme, and it displayed elevated levels of intra- and extracellular D-2-hydroxyglutarate oncometabolite with respect to the parental U-87 MG line. In addition, this cell line exhibits histone hypermethylation compared to the parental line. The IDH1 R132H isogenic cell model is a valuable in vitro cell-based tool for use in screening anticancer compounds for drug discoveries and development.

Therefore, the effect of Cyn has been also evaluated on the U-87 MG IDH1 R132H mutant glioblastoma cell line. Determination of IC50 values revealed that Cyn exhibited a strong cytotoxicity on IDH-mutant U-87 MG cells, resulting in 1.8, 4.2 and 3.4-fold lower, respectively, at 24, 48 and 72 h in comparison to the IC50 of Cyn on wild-type U-87 MG (Figure 1B and Figure 7B). Furthermore, a low concentration of Cyn 1 μM dramatically induced a cell morphological change already after 24 h of exposure, since the cells retracted their cell protrusions and became round-shaped (Figure 1C).

**Figure 8:**
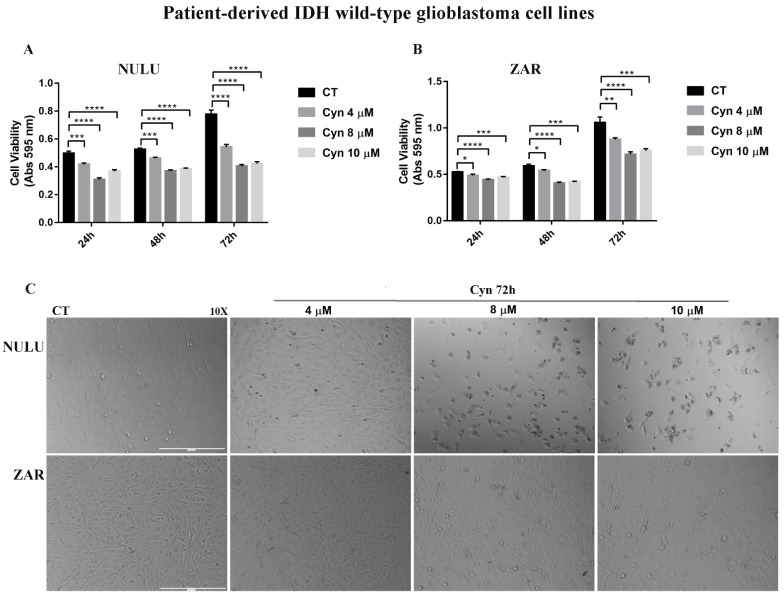
**Cytotoxic effects of Cyn on patient-derived glioblastoma cell lines NULU and ZAR.** (**A**) Cytotoxic effects of daily exposure to Cyn 4, 8 and 10 µM for 24, 48 and 72 h on NULU (**A**) and ZAR (**B**) cell metabolism. DMSO 0.1% was used as the vehicle control. (**C**) Morphological changes for the 4, 8 and 10 µM Cyn-treated NULU and ZAR cell lines for 24, 48 and 72 h. Data were analyzed by the Student’s *t*-test, *p*-value < 0.05. According to GraphPad Prism 7 software, * *p*-value 0.01–0.05 was considered statistically significant, ** *p*-value 0.001–0.01 very significant, *** *p*-value 0.0001–0.001 extremely significant and while **** *p*-values < 0.0001 were extremely significant. Taking together, these results clearly indicate the potentialities of Cyn as adjuvant therapy to conventional chemotherapy TMZ in IDH-mutant and wild-type glioblastoma.

Besides IDH1/2 mutations, the methylation status of the MGMT gene promoter is commonly used in the clinical diagnostic of glioblastoma as a predictive marker of sensitivity to TMZ. Therefore, the effects of Cyn were assessed on two patient-derived glioblastoma cell lines, NULU and ZAR, genetically characterized by IDH1/2 wild-type and unmethylated MGMT gene promoter, thus by a TMZ-resistant phenotype. Long-term treatment of NULU and ZAR cells lines with Cyn 4, 8 and 10 µM for 24, 48 and 72 h revealed that Cyn induced a time-dependent inhibition cell metabolism, as evidenced by the MTT assay (Figure 8A,B). Indeed, the cytotoxic effects of Cyn seemed to be more pronounced on the NULU cell line at 72 h of exposure than on the ZAR cell line, which also showed a remarkable change of the morphological shape (Figure 8C).

## 4. Discussion

Glioblastoma is one of the most aggressive tumors of the CNS, given the high infiltrating cell growth, marked angiogenesis and intrinsic or acquired drug resistance to chemotherapeutic agents. In glioblastoma, as in most neoplastic cells, all the homeostatic mechanisms to counteract the neoplastic transformation are altered. In fact, in glioblastoma, physiological strategies such as apoptosis and autophagy are deregulated, leading to uncontrolled tumor growth [34,35]. Cancer cells usually have higher intracellular ROS levels than normal tissue cells. ROS are extremely reactive molecules that regulate cell proliferation, contribute significantly to neoplastic transformation and are directly involved in the activation of the processes that control the growth of neoplastic cells. The redox imbalance resulting from the increase in ROS in the mitochondrial levels in the neoplastic cell could be responsible for the mitochondrial function, leading to cell death. Cancer cells are characterized by high levels of ROS due to an increased metabolic rate (Warburg effect) and hypoxia [36]. However, the upregulation of enzymatic and nonenzymatic antioxidant systems led cancer cells to develop a redox adaptation to excessive ROS [13]. These are common features to glioblastoma cells, since they present a high metabolic rate and, thus, high levels of ROS [21], and redox adaptation represents an essential factor in the resistance to oxidative stress-induced cell death [21].

Novel therapeutic strategies aim to increase the ROS levels to overcome the redox adaptation of GBM cells, inducing oxidative stress to trigger programmed cell death [37,38,39,40].

The role of SLs as ROS-inducing compounds has been widely accepted and correlated with their chemical structure [11,22,23]. Recently, the application of Cyn was revealed to be promising in the control of melanoma cell growth [10]. Similarly, in this study, we investigated the role of Cyn on glioblastoma cell proliferation. In U-87 MG cells, a continuous human glioblastoma cell line, a time-dependent IC50 of Cyn was estimated in the µM range, resulting in comparable with previously reported for melanoma cells [10]. These data clearly indicated a strong cytotoxic effect of Cyn on GBM cells. Indeed, the daily exposure of U-87 MG to Cyn 4, 8 and 10 µM, followed by cell counting and the MTT assay, confirmed the effects of Cyn in controlling cell proliferation and metabolism. A colony-forming assay further demonstrated the ability of Cyn to interfere with the U-87 MG clonogenicity, potentially blocking the GBM recurrence in vivo.

The drastic morphological change of Cyn-treated U-87 MG cells that retracted their protrusion, assuming a rounded shape, led to hypothesize the activation of programmed cell death pathways. Since preincubation with ROS scavenger NAC 3 mM reversed the Cyn-induced cytotoxicity and preserved cells from morphological change, the ROS levels were then measured. The concentrations of Cyn to induce oxidative stress in U-87 MG cells were chosen considering the effect of Cyn 8 µM on cell metabolism after 24 h of exposure and the reversion of cytotoxicity induced by the exposure of Cyn 25 µM by NAC. These concentrations led to measure the ROS level under chronic and acute toxicity in GBM cells. Qualitative and quantitative analyses revealed an increase in the ROS levels in U-87 MG cells treated with Cyn 8 and 25 µM. Considering the short half-life of ROS, an increase in the ROS levels, which is initially detectable after 2 h of exposure to Cyn 8 and 25 μM, remained visible only after 6 h of acute exposure to Cyn 25 µM. The downstream effect of oxidative stress induction is the loss of the mitochondrial membrane potential. Therefore, under acute toxicity, Cyn induced loss of the mitochondrial membrane potential in U-87 MG cells. After mitochondrial potential change, Cytochrome c was released as revealed by immunofluorescence staining; thus, the apoptotic cascade was activated. A decrease in pro-caspase 9, already visible at 24 h of exposure to Cyn 25 µM, was followed by the downstream reduction of pro-caspase 3 at 48 and 72 h of treatment, leading to a subsequent DNA fragmentation. The relevance of cell death pathways under Cyn-induced acute toxicity was confirmed by the significant increase in the LC3II/I ratio and concomitant reduction of autophagic substrate, p62.

Differently to acute toxicity, long but continuous inductions with low concentrations of Cyn 8 µM and, thus, with low levels of ROS drove cellular senescence in U-87 MG cells. Indeed, under chronic toxicity, Cyn 8 µM may represent a promising ROS-inducing compound able to control cell growth.

The effects of Cyn on NF-κB and the MAPK signaling pathways, which are among the most common dysregulated pathways in glioblastoma cells, have recently been reported. In accordance with the literature, Cyn 8 µM caused ERK dephosphorylation, accompanied by a relevant reduction of the NF-κB p65 subunit. This is not surprising, since the molecular docking calculations identified NF-κB as a potential target of Cyn and their chemical analogs [33].

Over the last two decades, the beneficial effects of phytochemicals in many human diseases, including glioblastoma, have been extensively demonstrated. In particular, the synergistic effects of natural compounds were demonstrated in combination with the radiotherapy (radiosensitization) and chemotherapy (chemosensitization) with conventional anticancer agents. Recently, the natural compound cinnamaldehyde has been proven to sensitize U-87 MG cells to the anthracycline doxorubicin [41]. The flavagline synthetic derivative, FL3, has been reported to trigger a senescence phenotype in combination with TMZ in both TMZ-responsive and -resistant glioblastoma cell lines [42]. However, despite the intense research effort, radio-chemotherapy with temozolomide remains the standard of care for glioblastoma treatment. Combination effects of Cyn and conventional chemotherapeutic drugs for esophageal cancer treatment, paclitaxel and 5-fluorouracil (5-FU) revealed that Cyn had synergism with both drugs [43]. Therefore, the combinatorial effect of Cyn with glioblastoma standard chemotherapy TMZ was investigated, resulting in synergistic cytotoxicity as evaluated by the Bliss additivity model.

Considering the high heterogeneity of glioblastoma as emerging from the deep molecular characterization of brain tumors by next-generation sequencing technology to integrate the histological analysis [44], the cytotoxicity of Cyn has been tested against different cell lines. In particular, cell lines here used were chosen according to the common predictive markers IDH 1/2 and MGMT gene promoter methylation [45,46]. In this regard, Cyn exhibited high cytotoxicity on the U-87 MG IDH1 R132H mutant glioblastoma cell line, the most common mutation in glioma, dramatically affecting the cell metabolism and morphology, with an IC50 lower than IDH wild-type U-87 MG. Interestingly, under the same conditions applied to treat U-87 MG cells, Cyn inhibited the cell viability of patient-derived glioblastoma cell lines NULU and ZAR, known to be characterized by the wild-type IDH status and by an unmethylated MGMT gene promoter and, thus, by a TMZ-resistant phenotype. These results underline the potentiality to translate this research to an in vivo study or a clinical trial in the next future. In fact, despite the strong cytotoxicity exhibited against wild-type and mutant IDH1 U-87 MG cells and patient-derived glioblastoma cells NULU and ZAR, Cyn has proven to selectively inhibit cancer cell lines and only weakly affect the proliferation of fibroblasts (Detroit 551 and explanted primary cells) and Chang liver cells [47]. Furthermore, recently, the cytotoxic effects of Cyn on the human dermal fibroblast cell line HDF compared to cancerous human esophageal carcinoma KYSE30, confirmed the selective toxicity of this sesquiterpene lactone and its anticancer potential [43]. This evidence represents considerable results of candidate Cyn as the adjuvant therapy to conventional TMZ chemotherapy in IDH wild-type and mutant glioblastoma.

## 5. Conclusions

Despite the intense research effort, a resolutive therapy for glioblastoma remains a challenge. Numerous clinical trials failed, due to the heterogeneity of glioblastoma; therefore, the Stupp protocol, that combines surgery with radio-chemotherapy with TMZ, represents nowadays the standard of care. However, the innate or acquired resistance of glioblastoma to TMZ treatment led to a chemoresistance phenotype and, thus, a poor prognosis for glioblastoma patients. The results here reported highlight the possibility to enhance the chemosensitivity of mutant and wild-type IDH glioblastoma to TMZ, using a natural substance cynaropicrin as the adjuvant therapy, potentially reducing the side effects due to the high dose of chemotherapy agent TMZ.

## Data Availability

The data presented in this study are available on request from the corresponding author.
